# Post heat shock tolerance: a neuroimmunological anti-inflammatory phenomenon

**DOI:** 10.1186/1476-9255-6-7

**Published:** 2009-03-27

**Authors:** Shahram Shahabi, Zuhair M Hassan, Nima Hosseini Jazani

**Affiliations:** 1 Department of Microbiology, Immunology and Genetics, Faculty of Medicine, Urmia University of Medical Sciences, Urmia, Iran; 2 Department of Immunology, School of Medical Sciences, Tarbiat Modarres University, Tehran, Iran

## Abstract

We previously showed that the progression of burn-induced injury was inhibited by exposing the peripheral area of injured skin to sublethal hyperthermia following the burn. We called this phenomenon post-heat shock tolerance. Here we suggest a mechanism for this phenomenon. Exposure of the peripheral primary hyperalgesic/allodynic area of burned skin to local hyperthermia (45°C, 30 seconds), which is a non-painful stimulus for normal skin, results in a painful sensation transmitted by nociceptors. This hyperthermia is too mild to induce any tissue injury, but it does result in pain due to burn-induced hyperalgesia/allodynia. This mild painful stimulus can result in the induction of descending anti-nociceptive mechanisms, especially in the adjacent burned area. Some of these inhibitory mechanisms, such as alterations of sympathetic outflow and the production of endogenous opioids, can modify peripheral tissue inflammation. This decrease in burn-induced inflammation can diminish the progression of burn injury.

## Introduction

We previously showed that it was possible to inhibit the progression of burn-induced skin injury by exposing the peripheral area of injured skin to sublethal hyperthermia following the burn [1]. In that study, second-degree burn injury was induced in mice, some of which had been injected with the opioid receptor blocker Naloxone 30 minutes prior to burn, and some of which were subjected to mild local hyperthermia (45°C, 1 and 3 minutes after burn). After 24 hours, local post-burn hyperthermia had decreased inducible nitric oxide synthase (iNOS) expression and tissue injury as assessed by the number of hair follicles. This effect appeared to be produced by endogenous opioid response [1].

Since burns occur due to lethal hyperthermia (lethal heat shock), and post-burn local hyperthermia (a slight and non lethal hyperthermia) helps to eliminate burn-induced injury, the reduction in injury due to post-burn local hyperthermia can be considered a kind of heat shock tolerance. Because this tolerance takes place after the heat shock (burn), the term "post-heat shock tolerance" seems appropriate [1]. Here we suggest a mechanism that explains how post-heat shock tolerance might inhibit the progression of burn injury.

## Hypothesis

Tissue necrosis progresses following a burn and is not limited to the time that the burn occurs [1-3]. The inflammatory response to this stress is one mechanism that can cause the progression of burn injury. By decreasing the inflammatory reaction following a burn, we might inhibit, at least partially, the progression of burn injury [3].

Neurogenic inflammatory responses contribute to burn-induced inflammation [4,5]. Noxious thermal stimuli to primary C-afferents lead to the release of various vasoactive sensory neuropeptides, (e.g., substance P), thereby contributing to local inflammatory events [4].

Burns are followed by the development of an area of hyperalgesia (and/or allodynia) around the lesion, which is known as the area of "primary hyperalgesia/allodynia". Surrounding this area, a zone of "secondary hyperalgesia/allodynia" appears in the undamaged skin and gradually increases in diameter with time. Hyperalgesia indicates a greater sensitivity to pain caused by a reduction in the pain threshold and an increase in the intensity of responses to supraliminal noxious stimuli; allodynia, on the other hand, is a painful sensation induced by normally non-painful, supraliminal noxious stimuli [6]. In the zone of primary hyperalgesia/allodynia, the stimulus/response function due to a thermal and/or mechanical stimulus is increased. In the area of secondary hyperalgesia/allodynia, there is hyperalgesia and/or allodynia only for mechanical stimuli, and not for heat [7].

The sensitization of the peripheral nociceptors is thought to be the neurophysiological mechanism underlying the hyperalgesia and allodynia to thermal stimuli that occurs at the lesion site. This phenomenon of sensitization to thermal stimuli has been observed not only in the peripheral afferents, but also at spinal, thalamic and cortical levels. These observations, however, might not imply an autonomous central process of sensitization, but might be effects of a potentiated peripheral input due to the sensitization of nociceptors [6].

Most nociceptive spinal cord neurons are inhibited tonically by descending inhibitory systems, which maintain control over the spinal cord. These neurons also are inhibited by heterotopic noxious stimuli, in line with the concept of diffuse noxious inhibitory control. This implies that painful stimulation at one site of the body might reduce pain at another site [8]. In fact, it has been observed that exposure of a defined tissue region to noxious stimuli engages a supraspinal loop, resulting in "heterotopic" activation of descending inhibition in other tissue regions (e.g., surrounding tissue) [9]. Various mechanisms are implicated for descending pain control including hard-wiring modes of communication and diffusion of neurotransmitters to sites distant from synaptic cleft. As it has been reviewed by Millan [9] among the mechanisms of descending pain control, alteration of sympathetic outflow and production of endogenous opioids can modify peripheral tissue inflammation and its associated nociception [9-13]. Sympathetic preganglionic nuclei of the thoracolumbar spinal cord receive an intense innervation from several classes of descending pathway, especially, those containing serotonin or norepinephrin, together with co-localised neuropeptides (e.g., substance P and thyrotropin releasing hormone) [9]. Opioidergic and noradrenergic systems have significant interactions on multiple levels [14]. At the peripheral level, the finding that the antihyperalgesic action of an alpha-2-adrenoceptor agonist was attenuated by a small dose of an opioid receptor antagonist injected into the inflamed paw suggests involvement of an opioidergic link in peripheral alpha-2-adrenergic actions [14,15]. Immune cells that release opioids in response to norepinephrine provide one potential link for an anti-hyperalgesic and anti-inflammatory interaction of opioid and noradrenergic mechanisms in the periphery [14]. Opioids can inhibit inflammation by stimulating opioid receptors on immune cells [16] and decreasing neurogenic inflammation by inhibiting the release of local pro-inflammatory neuropeptides (e.g., substance P) from nerve fibers [17-19].

**Figure 1:**
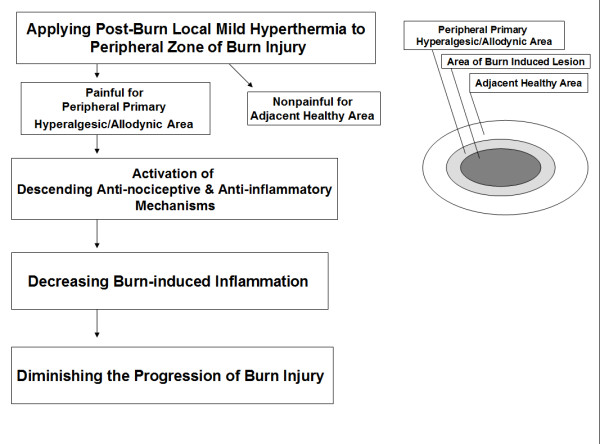
**The proposed mechanism for the protective effects of post-burn local hyperthermia against progression of burn-induced injury**.

Since there is hyperalgesia/allodynia in the peripheral zone of burn injury, it is likely that exposure of this area to local hyperthermia (45°C, 30 seconds), a non-painful stimulus for normal skin, results in a painful sensation transmitted by nociceptors (Figure 1). The cells of area exposed to hyperthermia should be so mildly injured that hyperthermia does not produce further sustained injury. On the other hands, as mentioned above, in the area of secondary hyperalgesia/allodynia, there is hyperalgesia and/or allodynia only for mechanical stimuli, and not for heat [7]. Therefore "peripheral zone of burn injury" refers to the margins of the area of primary hyperalgesia/allodynia.

In normal skin, heat resulting in painful sensation by nociceptors, carries a risk of tissue damage [6]; however, the hyperthermia used in post-heat shock tolerance is too mild to induce any tissue injury, but causes pain in the presence of burn-induced hyperalgesia/allodynia. This mild painful stimulus can result in the induction of descending anti-nociceptive mechanisms, especially in the adjacent burned area. As mentioned above, some of these inhibitory mechanisms (e.g., alterations of sympathetic outflow and production of endogenous opioids) can modify peripheral tissue inflammation [9]. Decreasing burn-induced inflammation can diminish the progression of burn injury (Figure 1).

## How can this hypothesis be tested?

Our previous findings support several aspects of this hypothesis:

1 – Post-heat shock tolerance decreased the expression of iNOS [1]. There is some evidence to suggest that NO might contribute to the development of the inflammatory response and secondary tissue injury following burns [3,20,21]. This is in agreement with the idea that inhibiting inflammation plays an important role in the mechanism of post-heat shock tolerance.

2 – When opioid receptors were blocked, post-heat shock tolerance did not decrease tissue damage [1]. This is in agreement with the idea that activation of the opioid system might underlie the decreased progression of burn injury in response to post-burn local hyperthermia.

Other parts of the hypothesis, however, still need to be tested. The following experiments could address key questions:

1 – If post-burn local hyperthermia activates descending pain control mechanisms, it should reduce the pain of burn injury. This could be tested by evaluating the anti-nociceptive effects of applying of post-burn local hyperthermia in some volunteers.

2 – If activation of the noradrenergic system is a mechanism by which post-burn local hyperthermia decreases the progression of burn injury, administration of adrenergic receptor antagonists before the application of post-burn local hyperthermia should inhibit the effects of post-burn local hyperthermia on the progression of burn injury.

## Competing interests

The authors declare that they have no competing interests.

## Authors' contributions

All authors read and approved the final manuscript.
